# Scalable Big Data Platform With End-to-End Traceability for Health Data Monitoring in Older Adults: Development and Performance Evaluation

**DOI:** 10.2196/81701

**Published:** 2025-12-22

**Authors:** Ander Cejudo, Yone Tellechea, Amaia Calvo, Aitor Almeida, Cristina Martín, Andoni Beristain

**Affiliations:** 1 Vicomtech Foundation, Basque Research and Technology Alliance (BRTA), Mikeletegi 57, Donostia-San Sebastián, Basque Country, 20009, Spain, 34 943 309 230; 2 Faculty of Engineering, University of Duesto, Bilbao, Spain; 3 e-Health Department, Biodonostia Health Research Institute, San Sebastián, Spain; 4 Computer Science and Artificial Intelligence Department, University of the Basque Country UPV/EHU, Donostia-San Sebastián, Spain

**Keywords:** older adults, data management, telemonitoring, early detection, wearable, big data

## Abstract

**Background:**

The increasing use of real-time health data from wearable devices and self-reported questionnaires offers significant opportunities for preventive care in aging populations. However, current health data platforms often lack built-in mechanisms for data and model traceability, version control, and coordinated management of heterogeneous data streams, which are essential for clinical accountability, regulatory compliance, and reproducibility. The absence of these features limits the reuse of health data and the reproducibility of analytical workflows across research and clinical environments.

**Objective:**

This work presents DeltaTrace, a unified big data health platform designed with traceability as a key architectural feature. The platform integrates end-to-end tracking of data and model versions with real-time and batch processing capabilities. Built entirely on open source technologies, DeltaTrace combines components for data management, model management, orchestration, and visualization. The main objective is to demonstrate that embedding traceability within the architecture enables scalable, auditable, and version-controlled processing of health data, thereby facilitating reproducible analytics and long-term maintenance of health monitoring systems.

**Methods:**

DeltaTrace adopts a medallion architecture implemented with Delta Lake to ensure atomic and version-controlled data transformations. Apache Spark is used for distributed computation, Apache Kafka for continuous data ingestion, and Apache Airflow for orchestration of batch and streaming workflows. MLflow manages the lifecycle and versioning of machine learning models, while Grafana provides visualization dashboards for real-time and aggregated data inspection. The platform is evaluated using continuous physiological signals from wearable devices and batch-ingested questionnaire data, combining synthetic and real data from the LifeSnaps dataset. Performance tests are conducted on central processing unit–only servers with 8-core and 24-core configurations to assess ingestion, aggregation, visualization, and anomaly detection latency.

**Results:**

DeltaTrace supports continuous processing for approximately 1500 users with end-to-end delays below 10 minutes. Ingestion and visualization tasks operate between mean 4.9 (SD 0.12) and 7.5 (SD 0.28) minutes, while aggregation and anomaly detection required less than mean 5.6 (SD 0.04) and 10.5 (SD 1.70) minutes, respectively. Increasing from 8 to 24 cores improved ingestion and cleaning latency by up to 25% and anomaly detection performance by up to 50%. The system maintains consistent performance across different data types, processing modes, and loads.

**Conclusions:**

DeltaTrace provides a scalable and modular architecture that incorporates traceability as a core component together with functions for model management, orchestration, and visualization. The platform enables complete version control across data and models and maintains performance under limited hardware conditions. These characteristics support reproducible and auditable health data processing and make DeltaTrace suitable for continuous monitoring and preventive health care in aging populations.

## Introduction

### Background

According to the World Health Organization, the number of individuals aged 60 years and older is expected to reach 2.1 billion by 2050, nearly doubling from 1.1 billion in 2020 [1]. This demographic shift is particularly pronounced in Europe, where persons aged 60 years or older are projected to account for 36% of the total population by 2050 [2]. Aging is associated with a higher prevalence of chronic and degenerative diseases, including cardiovascular conditions, diabetes, neurodegenerative disorders, and frailty syndromes. These often coexist, resulting in multimorbidity, which leads to increased health care demands, hospital admissions, and long-term care requirements [3,4].

Preventive care has become essential to address the growing health care burden in aging populations. Evidence suggests that interventions focusing on early detection, continuous monitoring, and timely management of health risks can significantly reduce complications, improve outcomes, and delay institutionalization [5,6]. Enabling older adults to remain at home with autonomy requires technical solutions that support preventive action through continuous observation and timely data-driven decisions.

Big data platforms have emerged as a relevant approach to support these goals. By integrating diverse data sources such as physiological signals from wearable devices [7] and electronic health records [8], these platforms can enable large-scale remote monitoring, predictive analytics, and personalized health care delivery [9]. Real-time processing, interoperability, and scalability are commonly addressed in many recent frameworks [10,11]. However, other critical features such as model traceability and data version control are often omitted [12-14]. These capabilities enable the tracking of each model inference and its underlying data, thereby supporting reproducibility and accountability.

Traceability is essential in health care applications, serving as an important process for recording data origins and the history of data generation and processing [15]. Within this domain, clinical decisions, audits, and regulatory compliance critically depend on knowing precisely which data, model, and processing logic were used at each stage of the data pipeline. This transparency ensures accountability and supports compliance requirements. However, despite this vital need, current solutions often prioritize data integrity during sharing but frequently lack transparency, traceability, and comprehensive integrity audits for cloud-stored medical data [16]. This absence of clear visibility can undermine patient trust and pose challenges related to computational power consumption and latency when sharing sensitive data across institutions [16].

### Related Work

Several studies have used big data technologies to analyze data for specific health conditions. The approach proposed by Ismail et al [12] developed a novel stream extract, transform, load (ETL) framework for real-time Twitter-based sentiment analysis using Kafka, Spark, Hadoop Distributed File System (HDFS), Hive, HBase, and Cassandra. Their experiments assessed performance across trigger intervals, dataset sizes, and storage technologies, ultimately finding HDFS to be the most effective storage solution. The authors reported increased ETL execution time when sentiment classification was integrated, highlighting trade-offs between system parameters and resource allocation. Similarly, Saeed and Saeed [13] used Apache Spark and Spark Streaming to analyze real-time hospital health care data for diabetes prediction. Their experiments found the gradient boosted tree classifier to be the most accurate, achieving 90.14% accuracy. The system was scalable and suitable for timely diagnosis, with future work aiming to incorporate additional data sources. Saleh et al [14] proposed TransformerFusionNet, a novel artificial intelligence (AI) model for real-time intensive care unit heart failure mortality prediction, achieving 91.72% accuracy. The model used Apache Spark and Kafka to stream and process structured and clinical note data, enabling scalable, real-time clinical decision support.

Beyond specific health conditions, other studies have proposed big data platforms designed for broader or more general applications. The Personal Health Dashboard introduced by Bahmani et al [10] used Kubernetes, Secure File Transfer Protocol, and serverless architectures to deliver scalable and secure health management using wearable, clinical, and omics data. Their experiments demonstrated dynamic scaling and presymptomatic COVID-19 detection, although further research was needed on omics data privacy. Similarly, Liu [17] developed a health data platform that calculated BMI from user-inputted height and weight, applied Chinese health standards, and provided personalized advice. While the core functionality was realized, the system remained preliminary, with ongoing work required to improve data integration and data quality.

A growing line of research has focused on data provenance, model management, and the reproducibility of machine learning (ML) pipelines in health care. According to Ahmed et al [15], data provenance frameworks play a crucial role in documenting data lineage and ensuring accountability in clinical decision systems; yet, they are often limited to metadata capture and do not scale effectively to large, heterogeneous health care datasets. The study by Belbasis and Panagiotou [18] highlighted that reproducibility and transparency remain major challenges for ML prediction models in health services research, emphasizing the need for comprehensive documentation of data, code, and analyses rather than focusing solely on large-scale processing. Xie et al [16] demonstrated the potential of MLflow for tracking model experiments in medical imaging tasks, but their work concentrated on model-level traceability rather than managing the entire data lifecycle. Finally, Mora-Cantallops et al [19] reviewed practices and data models for traceability in the context of building AI systems and observed that, despite the availability of many reproducibility tools, a common integrated approach for provenance and version control is currently lacking.

In summary, big data technology stacks have proven effective in delivering real-time, scalable health care solutions through advanced analytics and AI. However, existing solutions often lack a comprehensive approach that incorporates data traceability, including version control and lineage, critical requirements for health care applications. As summarized in Table 1 (see complete table in Multimedia Appendix 1), current systems also tend to treat key features such as model management, resource monitoring, data visualization, and distributed processing in a fragmented manner.

**Table 1.** Overview of recent studies using big data capabilities in health-related applications^a^.

Reference	Big data capabilities								
	Versioning	Scalability	Model management	Monitoring	Scheduling	Storage	Processing	Real time	Visualization
Ismail et al [12]					✓	✓	✓	✓	✓
Saeed and Saeed [13]							✓	✓	✓
Saleh et al [14]							✓	✓	
Ed-daoudy et al [20]		✓				✓	✓	✓	✓
Yıldırım et al [21]							✓	✓	✓
Ismail Ebada et al [22]							✓	✓	
Bahmani et al [10]		✓	✓	✓		✓	✓	✓	✓
Rashid et al [23]						✓	✓	✓	
Zheng and Ding [24]						✓	✓	✓	
Yongqiu et al [25]						✓	✓	✓	
DeltaTrace (ours)	✓	✓	✓	✓	✓	✓	✓	✓	✓

a Capabilities include versioning (data version control), scalability (scalability testing and handling large-scale data), model management (machine learning model management), monitoring (system or resource monitoring), scheduling (event or workflow scheduling), storage (distributed data storage), processing (distributed data processing), real time (real-time analytics support), and visualization (built-in data visualization tools). The final columns denote the primary task performed (eg, classification and anomaly detection) and the purpose or target condition addressed (eg, diabetes and general monitoring).

### Objective

This study addresses the lack of integrated data and model traceability in existing health data platforms, where version control and auditability are often treated as secondary features. The main objective is to design and develop a unified health data platform in which traceability is established as a central architectural principle, implemented together with essential components for scalability, model management, orchestration, and visualization, ensuring complete reproducibility and accountability throughout the data lifecycle.

The design of DeltaTrace is guided by research objectives that align with key big data capabilities identified in the literature and analyzed in the state-of-the-art review (Table 1). Specifically, the platform aims (1) to integrate versioning and traceability mechanisms to ensure full data and model lineage across the processing pipeline; (2) to support scalable storage and processing to handle continuous and batch data streams under variable system loads; and (3) to provide coordinated model management, scheduling, monitoring, and visualization within a single architecture to enable consistent and auditable workflows.

These objectives determined the overall architectural design, which is implemented using open source technologies chosen for their robustness, transparency, accessibility, and long-term sustainability. This approach ensures that the proposed platform not only addresses the limitations identified in existing solutions but also establishes traceability as a verifiable and reproducible property of the system architecture.

The paper is structured as follows. The Methods section describes the design of the DeltaTrace platform, detailing its key components, technologies, and evaluation setup. The Results section presents the main findings, including system performance, scalability, and visualization outcomes. The Discussion section summarizes the principal findings, interprets their clinical relevance, compares DeltaTrace with prior work, and outlines limitations and directions for future research. Finally, the Conclusions section highlights the main contributions and implications of DeltaTrace for reproducible and accountable health data management.

## Methods

### Design

DeltaTrace is designed to ingest, curate, transform, and store data from multiple heterogeneous sources, such as wearables and self-reported questionnaires, which differ in collection frequency, formats, and quality. The diversity of these sources requires a structured approach, motivating the use of an extract, load, transform (ELT) strategy and a medallion architecture to preserve raw data, enable flexible transformations, and maintain traceability throughout all processing stages.

The ETL approach often leads to irreversible transformations and potential loss of raw data. DeltaTrace adopts an ELT strategy, that is, raw data are first ingested and stored without alteration, ensuring full traceability and the ability to apply different transformation pipelines tailored to diverse processing or model input needs. This approach not only preserves original data but also supports reproducibility, facilitates auditing, and enhances compliance with the General Data Protection Regulation, which governs the processing of personal data in Europe, by supporting controlled and purposeful data transformations.

A key feature of the platform is end-to-end traceability. Every data transformation, model-related operation, such as training, evaluation, or inference, or change in the system is recorded, ensuring that the origin of each prediction or decision can be audited. This requirement is especially relevant for health care, where transparency is critical.

To address these needs, DeltaTrace is built upon the medallion architecture, a multilayered data structure that provides increasing levels of data quality, as recommended by Databricks. Each layer adheres to principles of atomicity, consistency, isolation, and durability (ACID), ensuring data reliability and recoverability. Data availability is ensured through the use of distributed and fault-tolerant components, such as Apache Spark and HDFS, which replicate data and store periodic checkpoints to enable recovery in case of system failure. Data accuracy is maintained by performing schema validation at ingestion, running automated data quality checks, and leveraging ACID transactions in Delta Lake to prevent corruption and guarantee consistency. Data usability is achieved through clear metadata definitions, versioned storage, and a configuration layer that enables data lineage tracking and reproducibility. By leveraging this architecture, DeltaTrace supports data governance, including availability, accuracy, and usability, across the entire data lifecycle. The quality of the data in each layer is described by the terms bronze (raw, ingested data), silver (cleaned datasets), and gold (enriched and aggregated data, ready for analytics and visualization). The medallion architecture does not replace other dimensional modeling techniques, and the schemas and tables within each layer can vary.

In addition to data engineering, DeltaTrace includes AI model lifecycle management. The platform supports all major steps in the model pipeline: data preparation, training, testing, and deployment. To enable full traceability of model versions, training data, and results, the system integrates MLflow [26], an open source MLOps platform. MLflow provides experiment tracking, pipeline versioning, and model serving. With MLflow, each model run is logged with detailed metadata, parameters, and outputs, enabling reproducible analysis and facilitating explainability by linking predictions to input features and model versions. Fairness auditing is supported through logging of model performance, while model metrics stored in MLflow enable continuous monitoring for potential data drift, alerting users to deviations in performance over time. These features allow the system to audit AI-driven decisions and evaluate the evolution of models over time, ensuring both transparency and reproducibility.

The automatic orchestration of data processing pipelines is essential to maintain the consistency and up-to-date status of the data lake. In the context of big data, 2 primary processing paradigms are used: batch processing and stream processing. The selection between these modes is driven by the nature and frequency of data collection. For instance, data collected at longer intervals, such as daily self-reports or weekly summaries, are typically handled through batch processing [27], which is well-suited for executing complex ELT tasks and periodic aggregations on larger volumes of data. Conversely, high-frequency data, such as minute-level readings from wearable sensors, are managed through stream processing. This model, implemented via Apache Kafka, enables real-time ingestion and processing by feeding data continuously into analytics components.

**Figure 1.**
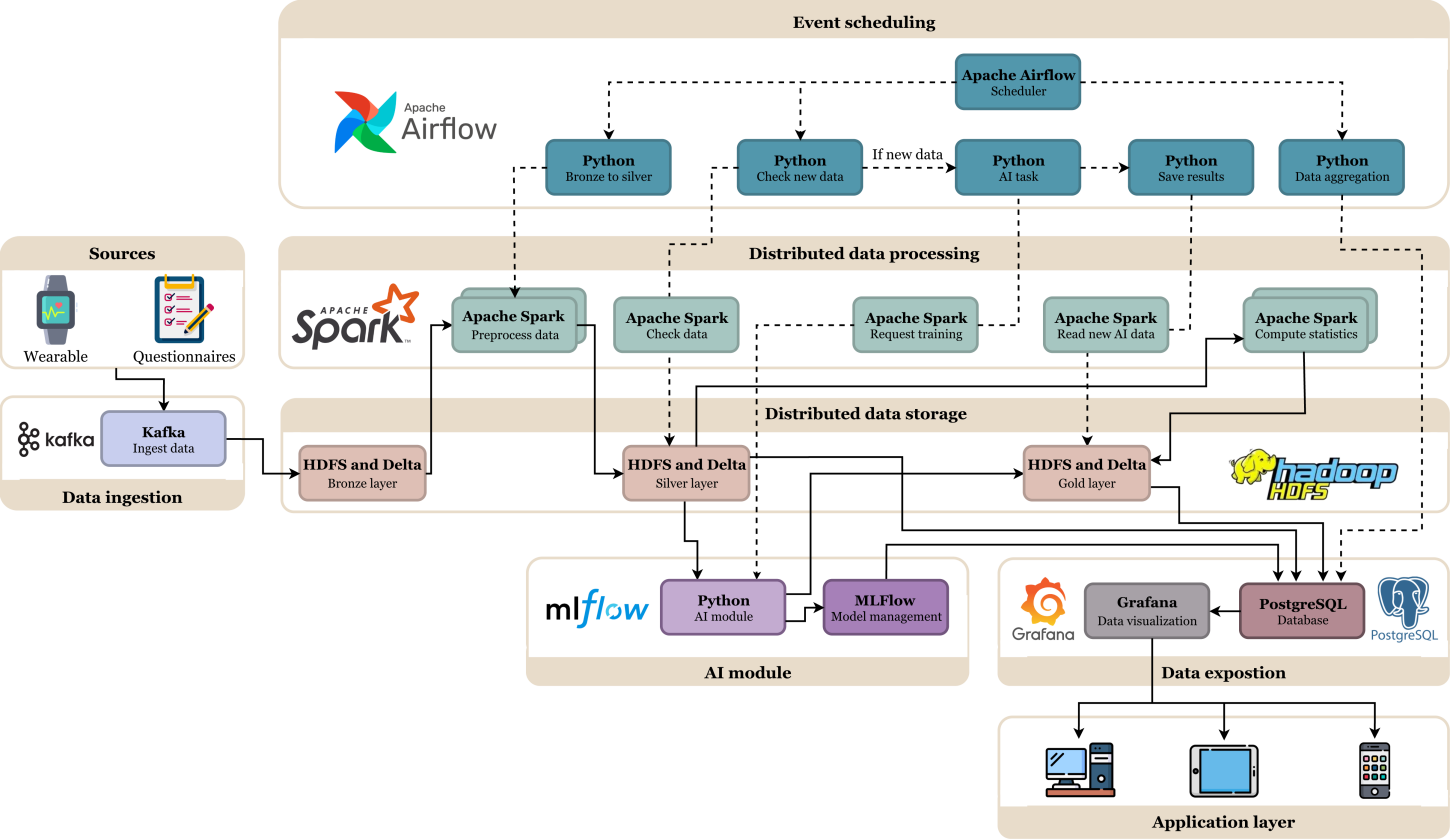
Schema of the DeltaTrace data platform. The continuous arrows indicate the direction of information flow, whereas the dashed arrows trigger the execution of a process. AI: artificial intelligence; HDFS: Hadoop Distributed File System.

All data-related workflows, including batch and real-time, are orchestrated using Apache Airflow [28], enabling flexible and robust task scheduling. Airflow manages the ELT pipeline across the different data layers, triggers model training when new data become available, and coordinates visualization updates and aggregation tasks. The high-level architecture of the DeltaTrace platform is presented in Figure 1. To support end-to-end data lifecycle management, from ingestion to visualization, the platform incorporates a comprehensive suite of open source technologies. The core components with the specific used software versions are described in Multimedia Appendix 2.

DeltaTrace has not only been built upon a carefully selected combination of open source technologies that support advanced analytics, real-time processing, and a high degree of traceability, but it has also been implemented alongside a dedicated configuration layer that enables full control of the data flow through simple configuration files. This layer abstracts operational complexity by allowing all core aspects, such as data source definitions, scheduling intervals, model parameters, and storage destinations, to be specified and modified without altering the underlying code. As a result, the platform is highly adaptable and maintainable, enabling rapid deployment and reconfiguration across different use cases or environments. More information about the configuration of DeltaTrace is provided in Multimedia Appendix 3.

All components in DeltaTrace are orchestrated to generate detailed logs to facilitate continuous monitoring and troubleshooting across the entire data processing pipeline. These logs record key operational events and system metrics, allowing users to verify the correct functioning of each component and trace the execution of data flows in real time. In addition, several of the integrated open source technologies inherently support data versioning and metadata management. For instance, intermediate results and model outputs are stored along with version identifiers and temporal metadata, enabling users to reconstruct historical states of the system or reproduce specific analyses. Checkpoints are also periodically generated to ensure fault tolerance and provide recovery capabilities, contributing to the overall transparency and reliability of platform operations.

### Data Ingestion

DeltaTrace has been designed to accommodate heterogeneous data sources, integrating both continuous sensor streams and periodic user-reported inputs. In the current implementation, 2 primary data sources are supported:

- Wearable devices: Data are streamed through 10 dedicated Apache Kafka topics, each corresponding to a specific measured variable (eg, heart rate, physical activity, or sleep). These topics receive both daily summaries and high-frequency time-series measurements, such as minute-level heart rate and detailed sleep stages.
- Self-reported questionnaires: Each questionnaire is made available to older adults through a dedicated web interface. Once completed, the responses are transmitted via a Representational State Transfer (REST) application programming interface (API) for further processing. These instruments are designed to assess various domains, including emotional well-being, motivation, and behavioral readiness. Specifically, the collected questionnaires include the State-Trait Anxiety Inventory (STAI), Positive and Negative Affect Schedule, Transtheoretical Model, and Behavioral Regulation in Exercise Questionnaire. These questionnaires are used to gather structured data and do not include unstructured text input, enabling consistent analysis across participants.

All the incoming data are continuously processed, providing real-time capabilities for data ingestion. Upon ingestion, the data are stored in the bronze layer of the Delta Lake storage system. This layer acts as an immutable repository of raw data, preserving the original structure and enabling flexible, reproducible processing at later stages. By maintaining the data in the original form, the system supports multiple downstream analyses and transformation strategies without imposing a fixed processing logic at the point of ingestion.

### Data Storage

#### Overview

DeltaTrace adopts a medallion architecture to organize and manage data across multiple layers (bronze, silver, and gold), each representing a progressively refined stage of data processing. This layered structure is implemented using Delta Lake, an open-source storage framework that brings ACID transaction support and scalable metadata handling to big data workloads. Delta Lake enables reliable data versioning, time travel, and schema enforcement. This architecture is further enhanced by AI-driven processes that operate on the curated data. AI models are trained using historical records and then deployed to perform real-time inference. Through this process, the AI layer transforms clean data into actionable insights, enriching the data pipeline and supporting decision-making. Each of the data layers is described below.

#### Bronze Layer: Raw Data

The bronze layer serves as the foundation, containing raw data ingested from multiple sources. These sources include wearable device data streamed through Kafka topics and questionnaire data collected via the REST API. For each source, a dedicated table is created to store the information in the original format, without structural modifications. This design preserves the integrity of the incoming data and supports traceability by ensuring that the unaltered raw inputs remain accessible for future processing or validation. Both wearable and questionnaire datasets follow a common structure that includes a user identifier and a date column, which enables seamless integration and cross-referencing between data types. This uniform schema allows data from heterogeneous sources to be combined efficiently for downstream analysis, as illustrated in Figure 1, where information from both wearables and questionnaires is jointly represented.

#### Silver Layer: Clean Data

The silver layer comprises data that have undergone refined cleaning operations aimed at improving consistency and usability. Typical tasks at this stage include the removal of duplicate records or the elimination of empty or irrelevant columns. These data support longitudinal and cross-sectional analyses, making them suitable for intermediate analytical workflows and for training ML models.

#### Gold Layer: Data Aggregations

The gold layer contains refined, domain-specific outputs tailored for end-user consumption. These include aggregated statistics, anomaly detection, and metrics intended to guide decisions made by clinicians, caregivers, or the older adults themselves. The gold layer is also the primary source for dynamic visualizations and alerts.

In parallel with this layered storage structure, a PostgreSQL (PSQL) [29] database is used to manage structured data essential for the proper functioning of the platform. This includes user registry information, authentication credentials, authorization roles, and other metadata required to support access control and administrative operations.

### Data Processing

Data processing is conducted in a distributed and scalable manner using Apache Spark, a widely adopted framework for large-scale data computation [30]. Spark enables parallel execution across multiple compute and storage nodes (Spark Workers), thereby facilitating the efficient transformation of high-volume datasets and ensuring that the system can scale horizontally by incorporating additional resources.

Each processing job operates independently and includes the necessary Delta Lake dependencies to support ACID transactions, schema enforcement, and versioning. Data are read from the appropriate Delta Lake table, transformed according to defined logic, and subsequently written to the next layer, maintaining full traceability throughout the process.

For each dataset stored in the bronze layer, a dedicated Spark job is defined and scheduled by Apache Airflow. These jobs are responsible for continuously reading newly ingested data, applying cleaning and normalization procedures, and writing the results to the corresponding silver table. To avoid redundant processing, Spark checkpoints are used to ensure that only new records are handled during each execution cycle. In addition to avoiding duplicate computation, checkpoints store essential metadata, including application configuration, the sequence of streaming transformations applied to the data, and information about incomplete batches, into fault-tolerant storage, enabling the system to recover easily from failures and maintain uninterrupted data processing.

The transition from silver to gold is also managed through Spark jobs. These processes may involve aggregations (eg, computation of means, counts, or other statistical metrics) or the preparation of data for ML inference. In cases where predictive analytics is required, the silver data are forwarded to the AI module, and the resulting outputs, such as model predictions or recommendations, are stored in the gold layer for downstream visualization and user interaction.

### Event Scheduling

DeltaTrace supports both batch and streaming modes for executing data processing tasks. In either case, data are ingested, transformed, and written to the appropriate storage layer (bronze, silver, or gold) according to the specific processing pipeline.

In the batch model, predefined tasks are executed at regular intervals, such as daily or weekly. This mode is suited for operations that do not require immediate responsiveness and benefit from scheduled, periodic processing. In contrast, the streaming model enables continuous ingestion and transformation of data as new records become available. Streaming pipelines are designed to operate persistently and are equipped with mechanisms to prevent duplication and handle errors, thereby ensuring data integrity.

All pipeline executions, both batch and streaming, are managed by the orchestrator of the platform, implemented using Apache Airflow. The orchestrator is responsible for scheduling task executions, monitoring resource use, logging events, sending error notifications, and automatically restarting pipelines in the event of failure. Streaming tasks are configured to run continuously and are automatically relaunched if they terminate unexpectedly.

Apache Airflow structures workflow execution using directed acyclic graphs (DAGs), where tasks are defined as nodes and dependencies as edges. DAGs provide a declarative mechanism to specify the execution order and relationships among tasks. Each task may consist of a discrete operation, such as an HTTP request or a Python script, or more complex jobs like Spark batch transformations or Kafka-based streaming listeners. The Airflow user interface enables visual monitoring and editing of these DAGs, facilitating clear and maintainable pipeline management.

### AI Module

The AI module is implemented as an independent component within the platform architecture, located between the silver and gold data layers. Designed as an optional step in the pipeline, it enables the transformation of clean silver-layer data into aggregated outputs, such as clustering or classification, for storage in the gold layer. This integration allows for the deployment of advanced analytics while preserving traceability and modularity. Although the MLlib library, built-in Apache Spark, provides capabilities for training models directly on DataFrames, a separate Python 3–based module has been adopted in DeltaTrace. This approach offers higher flexibility, allowing for seamless integration with MLflow for experiment tracking, support for a wide variety of models, and more comprehensive comparison and evaluation workflows.

The AI module is designed to support 1 or multiple AI pipelines capable of processing and inferring from the structured datasets available in the silver layer. To facilitate ease of use, the module exposes its functionality through a REST API developed using FastAPI. Two endpoints are provided:

- Train models: This endpoint orchestrates the data loading, preprocessing, and training procedures for all configured AI pipelines. Training metrics, model parameters and hyperparameters, and experiment metadata are logged to the MLflow tracking server. If a previous model exists for the same data source, its performance is compared to the newly trained model. If the new model achieves superior performance, this is registered as the latest version and made available for production.
- Predict: This end point initiates data loading, preprocessing, model retrieval, and inference stages. The system checks the model repository for a trained model corresponding to the input data source. If no model is available, an error is returned; otherwise, predictions are computed and returned as output.

Exposing the AI module as a REST service enables flexible integration with AI pipelines implemented in different programming languages. The AI models are stored in a centralized object repository, MinIO. In this case, a deep learning model based on recurrent neural networks has been selected for anomaly detection in wearable data [31]. Anomalies are defined as values that deviate from expected behavior, potentially triggering alerts to support preventive actions by health care providers. The chosen AI model is capable of learning temporal patterns in the data [32] to predict future values. These predicted values are then compared with the actual observations, and the resulting differences are modeled using a normal distribution. This approach enables the use of *z* scores to quantify deviations [33]. Depending on the magnitude of the deviation and predefined thresholds, anomalies are categorized into 3 levels: green (minor), yellow (moderate), and red (critical).

Model training and prediction tasks are orchestrated using Apache Airflow, which defines a pipeline composed of three primary stages: (1) checking for the availability of new data, (2) training or retraining the model, and (3) storing the resulting outputs. In the first stage, the orchestrator queries the Delta Lake silver layer to determine whether the volume of new data exceeds a predefined threshold for each data source. If this condition is met, an HTTP request is sent to the AI module containing identifiers such as the user_id, the variable to predict, and the relevant time range, serving primarily as a trigger for the AI module. Within the AI module, the corresponding data are loaded directly from Delta Lake based on these identifiers and processed to detect anomalies, and the results are then written to the gold table.

The anomaly detection component of DeltaTrace is based on predictive modeling using time-series data from wearable devices. The validation of these models included baseline, ML, and deep learning approaches for next-value prediction tasks, evaluated through user-based 3-fold cross-validation. The performance metrics included mean absolute error and root mean squared error. The selected model is required to achieve the lowest score among all evaluated models across these metrics and to demonstrate a clear improvement over the baseline approach. Based on these criteria, the attention-based long short-term memory (AttentionLSTM) model is selected for deployment due to its superior predictive performance. Details on the experimental setup, hyperparameter optimization, and model comparison are provided in Multimedia Appendix 4. Although the convolutional long short-term memory achieves a lower average mean absolute error for step prediction, the difference compared with AttentionLSTM is not statistically significant (*P*=.16, paired 2-tailed *t* test). Therefore, using the AttentionLSTM across all prediction tasks ensures consistency in the performance evaluation of DeltaTrace without compromising predictive accuracy. Anomalies are categorized using *z* score thresholds derived from the variability of the LifeSnaps dataset. To balance the 3 categories, we use *z*≥1.04 for green (minor), *z*≥1.29 for yellow (moderate), and *z*≥1.65 for red (critical). These thresholds also ensure that most anomalies remain minor, with fewer moderate and critical cases.

### Data Exposition

To support effective data visualization, selected outputs from the processing and inference stages are written in real time to a PSQL database. This database serves as a structured, query-efficient layer specifically designed to support time-series visualization tools such as Grafana.

Separate tables are created within the PSQL database to organize information by data type (eg, sleep, physical activity, and heart rate). Each table includes timestamp fields and primary keys, which facilitate time-series indexing, efficient joints, and fast query execution.

This architectural choice enables seamless integration with Grafana, allowing the platform to generate dynamic dashboards that display both real-time and historical data. Dashboards are configured to support multiple levels of analysis, including individualized monitoring for clinical care and aggregated views for population-level analytics. These visual interfaces enhance the interpretability of the system, supporting use cases such as anomaly detection, longitudinal health tracking, and cohort-based evaluations.

### Evaluation Design

To evaluate the performance and robustness of DeltaTrace, 2 complementary datasets are used: one synthetic dataset specifically generated for this study and one real-world dataset, LifeSnaps, selected for the richness and representativeness of multimodal health data. The combination of both datasets allows for a comprehensive evaluation, covering both controlled data generation scenarios and real-life variability, noise, and complexity.

- The synthetic dataset is created to simulate realistic health-related signals such as steps, heart rate, and sleep duration. To do so, first, the key variables to be simulated are identified and then established physiologically plausible value ranges for each of them, based on publicly available health guidelines and literature [34,35]. Data points are then generated with coherent temporal structure (eg, steps per day and heart rate per minute) using random sampling within the specified ranges. Finally, the simulated data stream is sent to Kafka, which ingests the information into the bronze layer of DeltaTrace for further processing. Performance results are reported in terms of requests per second, where a request is defined as the ingestion of a single record for a given topic (eg, 1 temperature value per second or 1 heart rate measurement per second). Each simulated user includes multiple wearable-derived data sources, with up to 10 variables (topics) captured concurrently, assuming a frequency of 1 record per second. This dataset enables the generation of all necessary records in a controlled manner, allowing scalability tests to be conducted without constraints on data volume.
- The second dataset is LifeSnaps [36], a publicly available, multimodal, longitudinal dataset collected over approximately 4 months from 71 participants across Europe. It integrates high-resolution wearable data, ecological momentary assessments, and validated surveys, resulting in over 35 distinct types of measurements captured at temporal granularities ranging from seconds to days. The wearable device used for data capture is a Fitbit Sense, which includes readings from vital signals such as heart rate, sleep patterns, steps, blood oxygen saturation, temperature, and stress-related metrics. Additionally, ecological momentary assessment data encompass contextual and mood surveys, while traditional survey instruments cover personality, motivation, and emotional states. Altogether, the dataset comprises over 71 million rows, allowing comprehensive evaluation across physiological, behavioral, and psychological health dimensions.

For each experiment, performance is evaluated in terms of computational time as the number of requests per second increases. This process, known as a stress test, is conducted using the simulated dataset, while visualizations are produced with the LifeSnaps dataset.

The performance testing server for DeltaTrace is a Linux-based Ubuntu system with a 5.15 kernel, powered by dual Intel Xeon Gold 5218 central processing units (CPUs; 24 cores total at 2.30 GHz). It has 64 GB error-correcting code RAM, 8 GB swap, and a multilevel cache (L1: 256 KB and L2 and L3: 32 MB each). The server runs in a kernel-based virtual machine virtualized environment. Storage includes a 500 GB primary disk, a 60 GB secondary disk, and several small loop devices.

### Ethical Considerations

This study did not involve any direct physical or mental intervention with participants. All data used were either synthetic, generated based on reference values reported in the literature, or obtained from LifeSnap, a publicly available dataset. Thus, this study did not require ethics approval, as it did not involve human participants or personal identifiable information.

## Results

### Silver Layer: Clean Data

**Figure 2.**
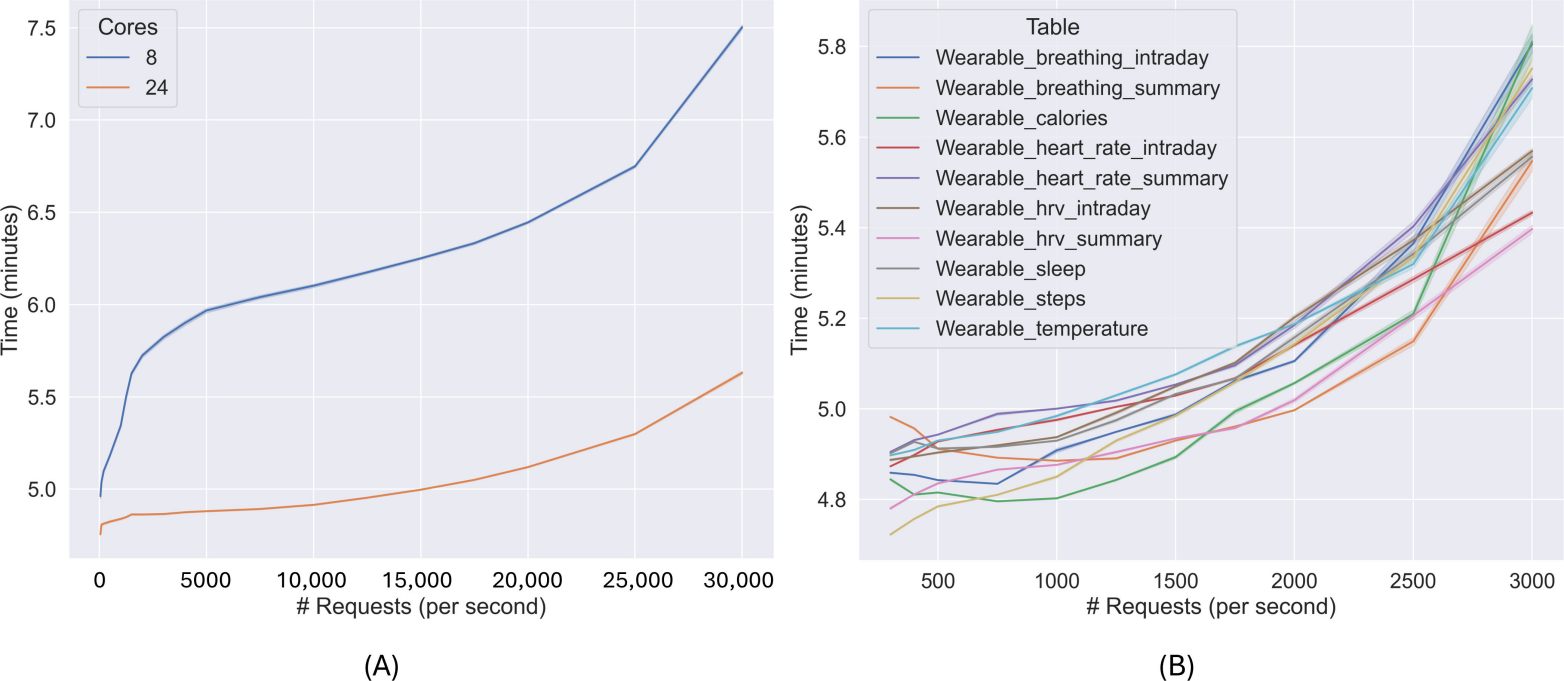
Time needed to process raw data from generation to the silver layer and make it available for visualization. (**A**) Time from data generation to silver layer and (**B**) time required for each data topic to be processed and available in the silver layer.

In this set of experiments, the stress test is run and assessed until the clean data are available for visualization and consultation. The objective is to assess whether DeltaTrace is capable of handling high-throughput scenarios as demand scales. In addition, real data are visualized to show the system output. The results of the stress test are presented in Figure 2, where the x-axis represents the number of requests per second, each request corresponding to a single record of wearable data, such as a heart rate measurement, and the y-axis shows the processing time in minutes.

Figure 2A shows the time required to process data from the moment they are sent until they become available in the silver layer (y-axis), for different CPU configurations (8 and 24 cores) and increasing request rates (x-axis). At 50 requests per second, the 8-core setup requires a mean of 5.1 (SD 0.2) minutes, while the 24-core setup requires a mean of 4.9 (SD 0.12) minutes (a difference of 0.2 minutes). At 15,000 requests per second, the mean time increases to 6.3 (SD 0.14) minutes (8 cores) and 5 (SD 0.06) minutes (24 cores), with a difference of 1.3 minutes. At 30,000 requests per second, the mean values reach 7.5 (SD 0.28) and 5.6 (SD 0.19) minutes, respectively, with a difference of 1.9 minutes. These values correspond to an improvement of 4.15%, 20.05%, and 24.95%, respectively, when using 24 cores compared to the 8-core configuration.

Figure 2B provides a more detailed view by showing the processing time per data topic sent by the wearable device using the 24-core setup. When 500 requests per second are received, the time ranges between 4.7 and 4.9 minutes depending on the data topic. At 1500 requests per second, the range increases slightly (4.9 to 5.1 minutes). At 3000 requests per second, the time ranges from 5.4 to 5.8 minutes. This indicates a maximum difference of 25 seconds between the fastest and slowest topics (processed simultaneously), which reflects consistent behavior across data streams.

**Figure 3.**
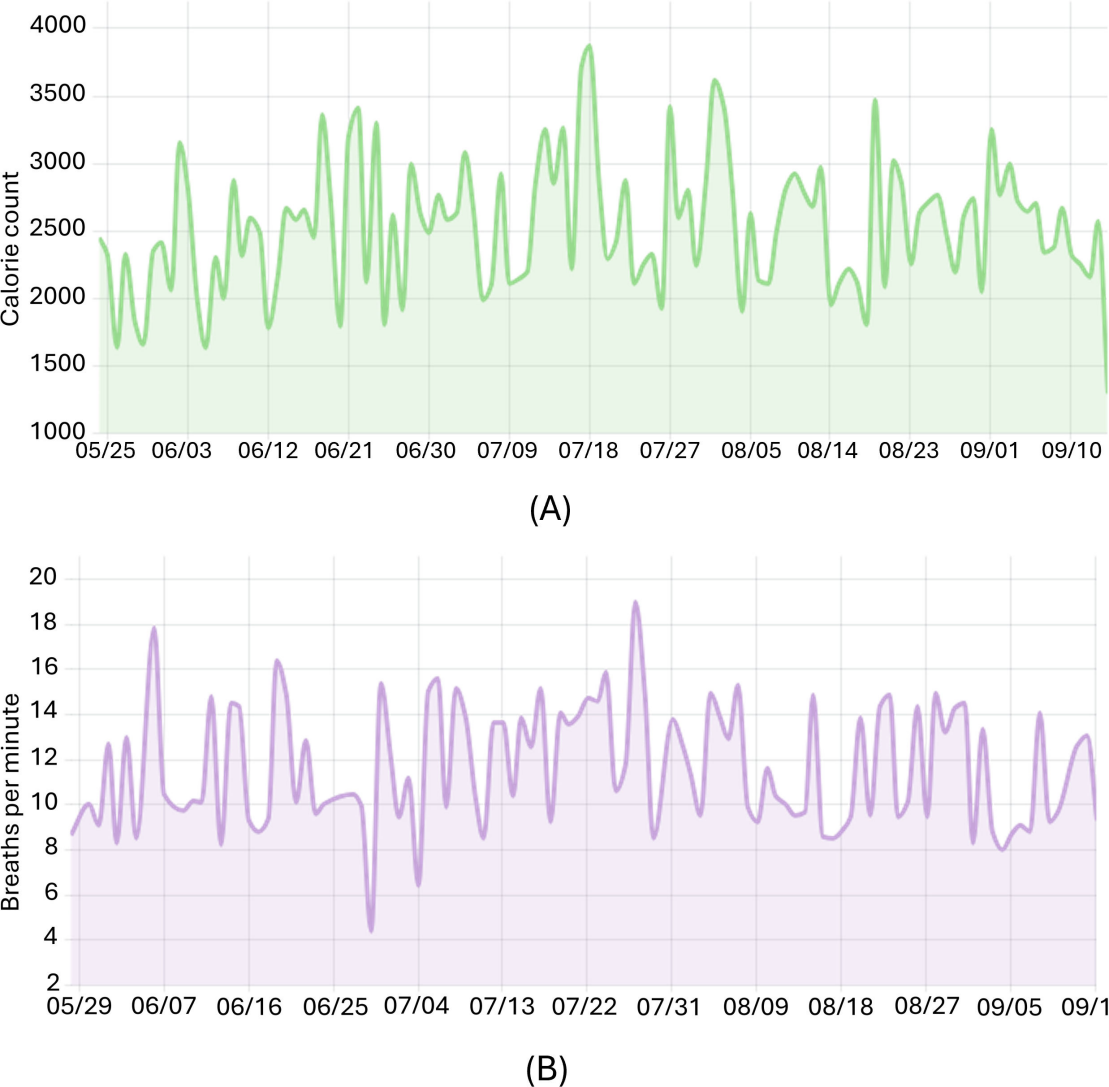
Data received from wearable devices, ingested into the platform, and cleaned in the silver layer. (A) Daily calorie consumption and (B) number of breaths per minute.

In Figure 3, actual data from the LifeSnaps dataset are shown using Grafana. In Figure 3A, the x-axis represents the date of the record, and the y-axis shows the calorie count, while in Figure 3B, the y-axis represents the number of breaths per minute. The data are plotted in real time, with an observed delay of approximately 4.6 minutes when 71 users are monitored. Two data sources are shown for illustration purposes: Figure 3A presents the number of calories burnt throughout the day, and Figure 3B shows the number of breaths per minute, both collected over several days.

In summary, the computational cost as the platform load increases is evaluated, with the 24-core configuration being up to 24.95% more efficient than the 8-core configuration. The time measurements include the full processing pipeline (from data ingestion to visualization at the silver layer). Additionally, the time difference between data topics remains within 25 seconds under all tested loads, indicating uniform performance across heterogeneous sources. These results confirm that DeltaTrace maintains consistent latency and scales proportionally with the number of requests for data to be available for visualization and consultation.

### Gold Layer: Data Aggregations

**Figure 4.**
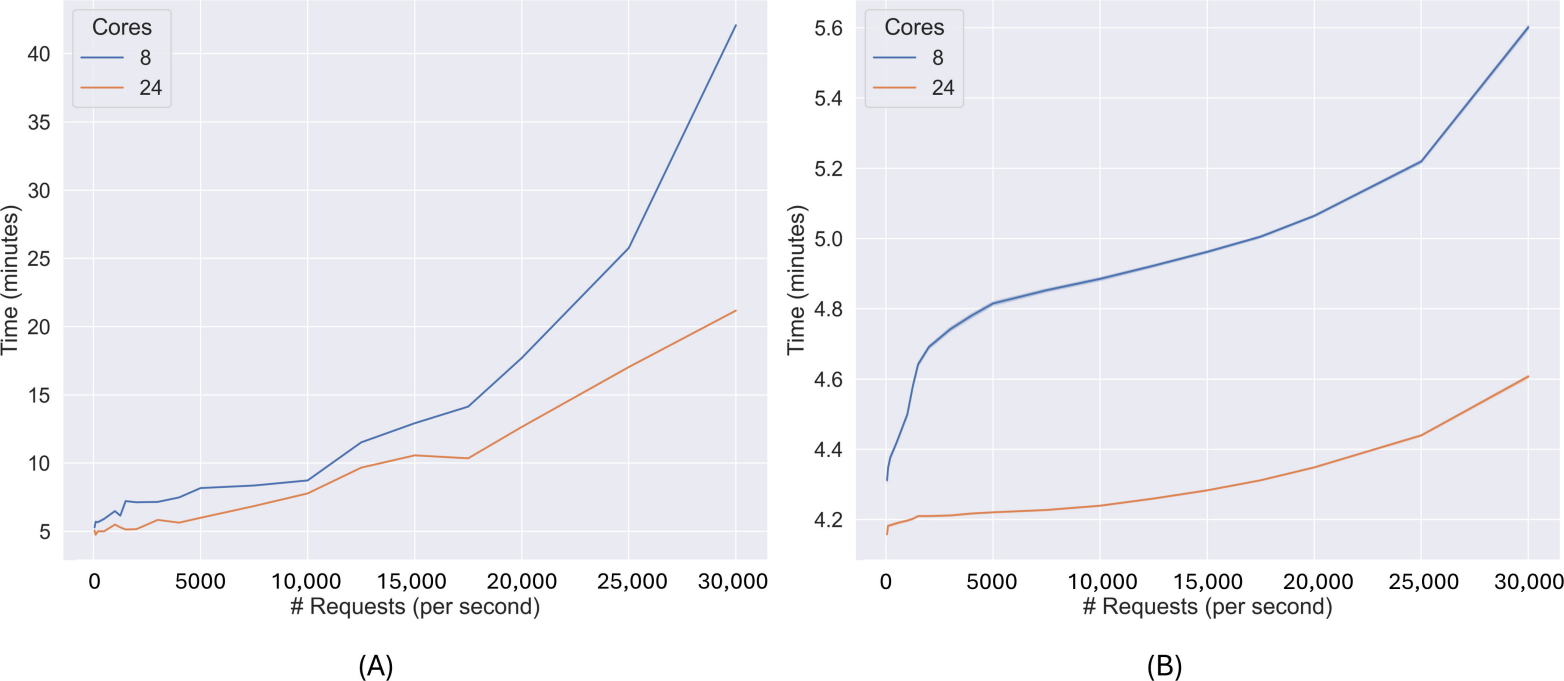
Time required to process raw data from generation to plotting in the gold layer for both anomaly detection and statistical analysis, comparing 8- and 24-core configurations under increasing request rates. (A) Time from data generation to anomaly detection (gold) and (B) time from data generation to statistical analysis (gold).

In this second set of experiments, the efficiency of DeltaTrace in performing aggregations over clean data under increasing loads is evaluated. The goal is to assess system performance when a real-time AI model and statistical aggregation processes continuously read data from the silver layer and write results to the gold layer. Computational time is measured under 8- and 24-core CPU configurations, as the request rate increases. In Figure 4, the x-axis represents the number of requests per second, while the y-axis indicates the processing time in minutes. The performance of the AI model is detailed in Multimedia Appendix 4.

As shown in Figure 4A, the time required from the moment the data are sent until anomalies are generated and made available for visualization is represented on the y-axis, with the request rate shown on the x-axis. At 50 requests per second, the difference between the 8- and 24-core settings is 0.2 minutes. This difference increases to 2.1 minutes at 1500 requests per second and to 2.4 minutes at 15,000 requests per second. At 30,000 requests per second, the difference reaches 20.9 minutes. These results correspond to improvements of 3.78% at 50 requests per second, 18.58% at 15,000 requests per second, and 49.67% at 30,000 requests per second when using 24 cores compared to the 8-core setup. The 24-core configuration also consistently exhibits lower SDs, indicating more stable performance under all tested loads.

In Figure 4B, the processing time required to compute and visualize statistical aggregations (such as mean values) is shown. At 50 requests per second, the 24-core configuration processes data 0.1 minutes faster than the 8-core setup. This difference increases to 0.4 minutes at 1500 requests per second and to 0.7 minutes at 15,000 requests per second. At 30,000 requests per second, the difference reaches 1 minute. These values represent improvements of 8.62%, 14.11%, and 17.86%, respectively, when using the 24-core configuration.

**Figure 5.**
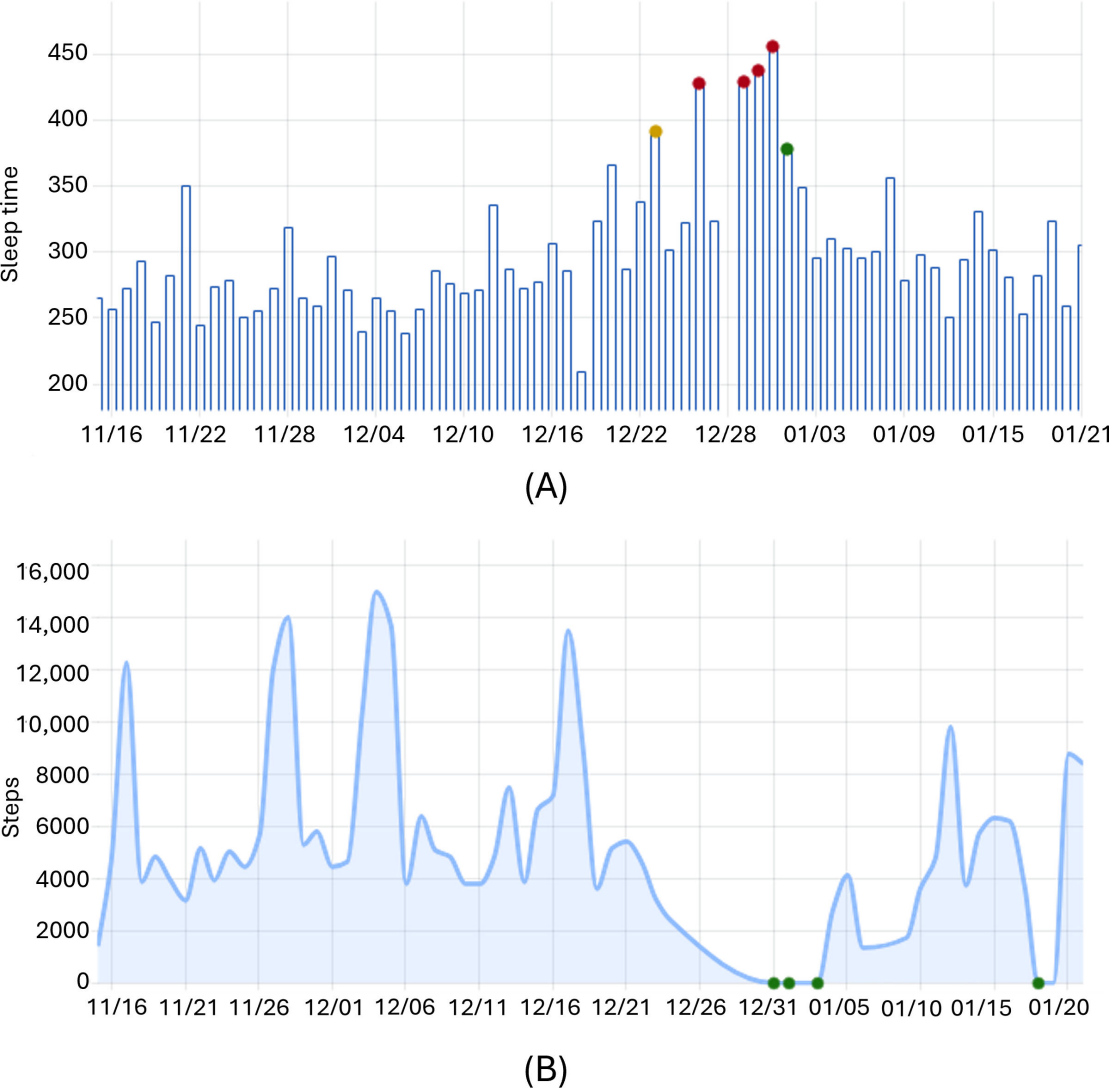
Visualization of behavioral anomalies in wearable data based on sleep and step patterns. Anomalies are marked in color: red (critical), yellow (moderate), and green (minor), indicating deviations from the typical pattern. (A) Sleep duration over time, measured in minutes with the detected anomalies and (B) step count per day with the detected anomalies.

**Figure 6.**
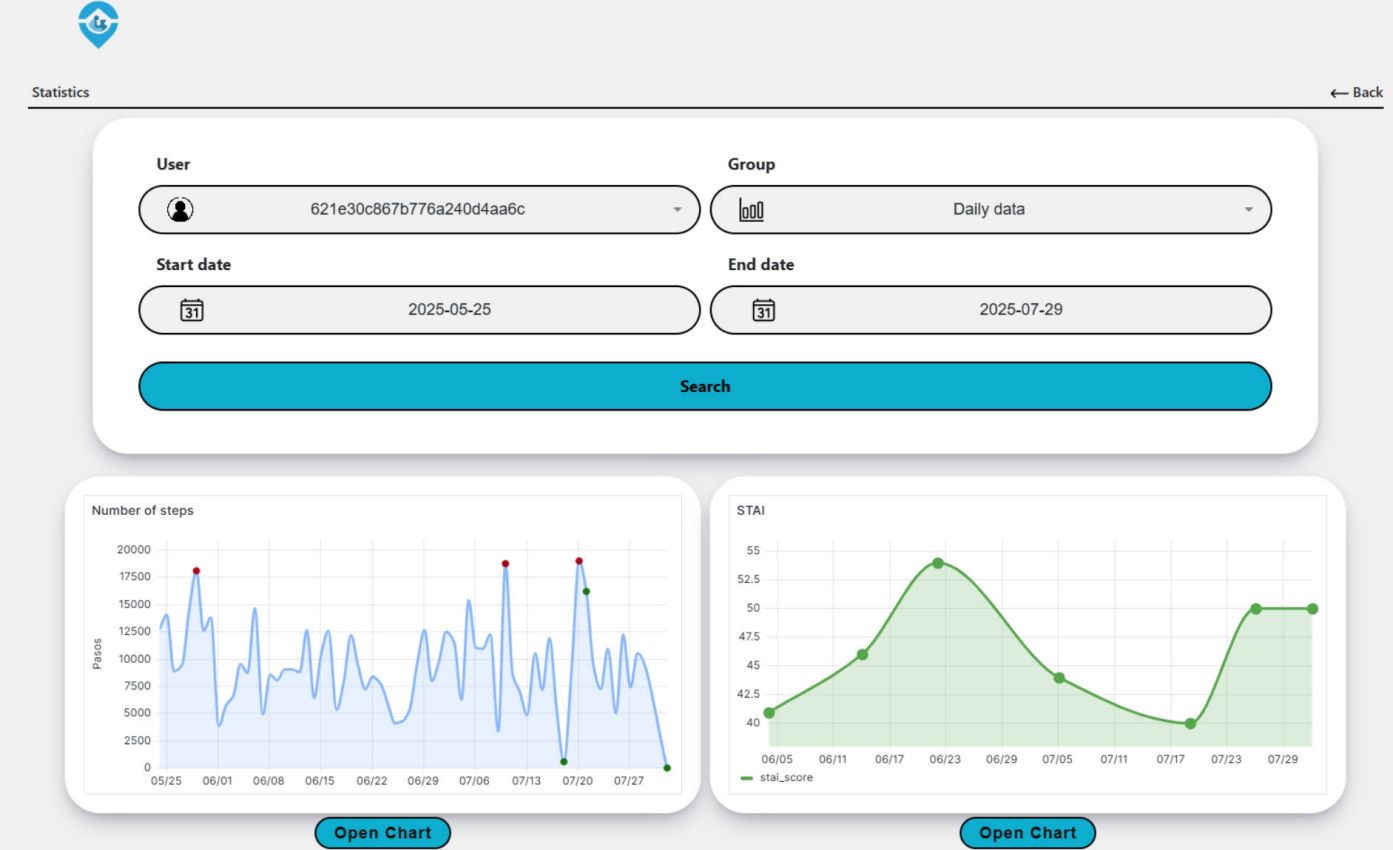
Web application for health care professionals enabling user selection, visualization grouping, and date filtering. The left panel displays daily step count for the selected user, highlighting anomalies by deviation severity: red (critical), yellow (moderate), and green (minor). The right panel shows the reported score for the anxiety trait from the State-Trait Anxiety Inventory scale.

The anomalies detected in the LifeSnaps dataset are presented in Figure 5. Figure 6A displays sleep duration (in minutes) for a single user, with anomalies marked in color based on the level of deviation from learned user patterns. In this case, consecutive anomalies appear between December 23, 2021, and January 1, 2022, with 1 yellow anomaly followed by 4 red and 1 green anomaly, all corresponding to unusually long sleep durations. In Figure 5B, step count data show 4 green anomalies identified as potential inactivity events, which could also be attributed to not wearing the wearable device during those days.

In addition, a web application for health care professionals has been developed, as shown in Figure 6, being part of the application layer in Figure 1. Grafana panels are embedded for visual analysis and are continuously updated as new data arrive, with delays indicated in Figure 4. The left panel displays anomalies detected in daily step counts, while the right panel shows scores for the STAI anxiety trait. Unlike sensor data, STAI questionnaires are received at irregular intervals and processed in batches through tasks scheduled in Apache Airflow, which optimizes resource use for low-frequency sources. This configuration enables visual comparison between anomalies in activity data and self-reported questionnaire results.

In summary, this set of experiments presents the computational time required to aggregate real-time data and make it available to health care professionals. When the request rate is 15,000 per second, statistical aggregations are available in approximately 4.3 minutes, whereas the anomaly detection model requires 10.5 minutes, times that are sufficient for several clinical use cases [37-45] and consistent with those reported in previous studies of similar big data platforms [10,46]. At lower request rates (below 15,000 per second), anomalies are computed in 5 to 10 minutes after the data are sent. However, at 30,000 requests per second, anomaly detection latency exceeds 40 minutes in the 8-core configuration, compared to 20 minutes in the 24-core setup. This nonlinear increase suggests saturation in the lower-resource configuration, with the latency line showing a marked nonlinearity (saturation) starting around 20,000 requests per second. This relationship is fitted by a quadratic model (polynomial regression, adjusted of 0.98 for 8 cores and 0.99 for 24 cores), confirming a significant nonlinear relationship (*P*<.0001 for the quadratic term). In contrast, statistical aggregations remain under 5.6 minutes across all loads, indicating more stable and scalable performance. These results highlight the need to allocate sufficient computational resources depending on the expected load and latency requirements for data to be available for visualization and consultation.

## Discussion

### Principal Findings

The experimental results demonstrate the performance of DeltaTrace across different data processing layers and system loads. In the first set of experiments, which focuses on the processing time from data generation to the silver layer, the platform can maintain mean processing times between 4.9 (SD 0.12) and 7.5 (SD 0.28) minutes depending on the request rate and data topic. The 24-core configuration shows improvements of up to 24.95% compared to the 8-core configuration. While perfect scaling would yield an improvement of 66%, this is unrealistic since part of the workload is not parallelizable. According to Amdahl’s law [37], an efficiency of 80%‐85% would typically produce 25%‐40% improvement, consistent with our observation. In all cases, the delay between different data sources remains approximately 25 seconds, which confirms the consistency of the system across heterogeneous input streams.

In the second set of experiments, which evaluates the processing time to deliver statistical aggregations and anomaly detection in the gold layer, the computational demand of anomaly detection is higher than that of simple aggregations. With 15,000 requests per second, the time required for statistical output is approximately 4.3 minutes, whereas the time for anomaly detection reaches 10.5 minutes. At 30,000 requests per second, the 8-core configuration exceeds 40 minutes for anomaly detection, while the 24-core setup remains under 20 minutes. Statistical aggregations, in contrast, remain under 5.6 minutes in all configurations and scenarios, showing predictable and stable behavior.

These results demonstrate that DeltaTrace maintains stable performance and predictable latency under increasing data loads, confirming the scalability of the proposed architecture when limited computing resources are available. Beyond performance, the experimental evaluation confirms that the platform design successfully fulfills the defined research objectives. The implemented mechanisms ensure versioning and traceability across all data processing layers, stable scalability under varying loads, and consistent operation of model management, scheduling, and monitoring components. These results demonstrate that DeltaTrace does not only integrate well-known technologies but also applies them within a coherent architecture that operationalizes traceability as a core and verifiable property of a health data platform. The real-time monitoring capabilities and traceability features evaluated in these experiments have direct implications for clinical applications, which are further elaborated in the following subsection.

### Clinical Implications

DeltaTrace provides a transparent and reproducible foundation for clinical applications where accountability and data integrity are essential. By allowing clinicians to trace each alert or output back to its source data, processing pipeline, and model version, the platform builds trust in automated decision support. This is especially important when clinical actions depend on system-generated insights. The ability to monitor trends in sleep, mobility, or heart rate in near real time can support early identification of health deterioration, enabling more proactive care for older adults. These features make DeltaTrace a strong candidate for integration into remote patient monitoring programs and digital health strategies focused on prevention and early intervention.

In this work, the platform has been intentionally evaluated using a minimal hardware configuration to establish a lower bound on the resources required to provide near real-time processing. Under these conditions, DeltaTrace can detect and process anomalies for approximately 1500 users in under 10 minutes. This response time is suitable for a wide range of clinical and health monitoring applications where reactions within this time frame are beneficial. These include chronic conditions such as sleep apnea [38], hormonal cycle irregularities [39], or psychiatric events that evolve over time [40-42]. In contrast, other conditions, such as stroke, anaphylaxis, or cardiac arrest [43-45], require faster interventions, often within minutes. In such cases, additional resources (eg, graphics processing units [GPUs] for inference and multiple server nodes) can be deployed to reduce latency. Similar processing delays (5 to 10 minutes) have been reported in previous studies [10,46].

### Comparison With Prior Work

Previous work has reported enhanced efficiency with Apache Spark and HDFS if more hardware resources are provided [47,48]. As reported by Sewal and Singh [49], the execution time can decrease from 1019 to 382 seconds if 5 nodes are provided, which implied an improvement of 73.51%. Similarly, evaluations reported by Buber and Diri [50] have shown that GPU-based processing can accelerate execution by a factor of 4 to 5 compared to CPU-only configurations. These enhancements can be achieved without requiring modifications to the software architecture, thereby maintaining consistency, traceability, and scalability across different deployment environments.

In this paper, a separate AI module with API-based communication is proposed for detecting anomalies in wearable data. This is a general design that provides a significant benefit, as the AI process is decoupled, and any AI task can be included in the pipeline without modifying the architecture, such as classification and clustering tasks. The MLflow framework allows the logging of any type of model, including ML and deep learning, and also state-of-the-art models such as large language models and transformer-based models. The software layer implemented on top of this architecture allows the evaluation of the performance when the load increases, being able to identify if the hardware allocation is proper for the specified AI task and use case. This design contributes to open challenges [51] and current trends [52] identified by previous reviews, especially with deep learning models such as recurrent neural networks, regarding model scaling, distributed computing, and high-scale data processing, all of these while keeping traceability features.

DeltaTrace also contributes to ongoing initiatives such as the European Health Data Space (EHDS) Regulation, which aims to establish a common framework for the secure use and exchange of health data within the European Union. Traceability and data governance are among the core pillars of this regulation [53-55]. The platform provides full traceability, including version control for data, models, and code, as well as detailed tracking of all transformations applied after data ingestion. These capabilities directly support EHDS requirements for auditability and transparency in both primary and secondary uses of health data [56,57]. In addition, the system integrates model management, resource monitoring, and real-time analytics under a unified architecture based on open source components, aligning with the EHDS objective of promoting trusted and scalable health data infrastructures [58]. These characteristics position DeltaTrace as a suitable technical foundation for future EHDS-compliant deployments.

This alignment with EHDS is particularly valuable when comparing DeltaTrace with previous work, as most existing platforms focus primarily on large-scale data processing, real-time analytics, or specific task performance but provide limited support for traceability, versioning, model management, and comprehensive orchestration. As shown in Table 1, many prior studies omit key mechanisms for tracking data and model transformations, monitoring system resources, and ensuring reproducibility across the pipeline. In contrast, DeltaTrace integrates end-to-end versioning of datasets and models through MLflow, robust orchestration and workflow scheduling via Apache Airflow, scalable processing of batch and streaming data with Apache Spark, and fault-tolerant storage and metadata management with Delta Lake. By combining these capabilities, DeltaTrace ensures traceability, auditability, and reproducibility, providing a more comprehensive and transparent framework for health data management and a stronger technical foundation for EHDS-compliant deployments.

### Limitations

The evaluation of DeltaTrace was first conducted in a single-server environment to establish a controlled baseline and analyze system performance under stable and reproducible conditions. Within this setup, experiments were executed using constant data input rates and a fixed anomaly detection model, which simplified testing and allowed for consistent performance assessment. This configuration may not fully reflect the variability and operational challenges present in multinode or real-world deployments. In the current implementation, the AI inference module operates as a standalone Python service, which may limit scalability compared to the distributed data processing components. However, the modular architecture based on containerized services allows future extensions toward distributed or parallel inference using orchestration frameworks such as Kubernetes. This design intentionally separates the AI inference module from the distributed data layer to enable flexible integration of custom deep learning pipelines. For the present implementation, these distributed capabilities have not been tested, and this represents a limitation of the current proposal. Moreover, the anomaly detection model cannot, in its current form, distinguish between true behavioral anomalies (eg, inactivity) and data quality issues (eg, sensor nonadherence or device-off). Additionally, aspects such as human interaction and workflow integration were not examined. Despite these constraints, the obtained results provide a solid foundation for understanding DeltaTrace behavior under controlled conditions and for guiding future evaluations in larger and more dynamic environments.

### Strengths and Future Work

All technologies composing the platform are open source, offering transparency, adaptability, and long-term sustainability. This choice also supports future work focused on enhancing the security and interoperability mechanisms of the proposed big data platform [59]. Core components such as Kafka, Delta Lake, MLflow, and Airflow include built-in features such as encrypted data transmission (Transport Layer Security), encrypted storage (Advanced Encryption Standard), role-based access control, and execution logging, which are integrated into the platform. Data processed throughout the system are consistently associated with anonymized identifiers, used to securely match heterogeneous sources such as questionnaire responses and physiological signals. These identifiers are also used by the AI module to construct temporal sequences and apply sliding window techniques across unified data streams. Although accountability, data integrity, and traceability are demonstrated through system mechanisms and workflow examples (Figure 1 and Multimedia Appendix 3), their empirical validation under real deployment conditions remains part of future work.

Future work will include testing the platform in distributed and GPU-accelerated environments to further assess performance and responsiveness under higher data volumes. Further evaluations will also involve human-in-the-loop scenarios to examine usability and workflow integration in operational health settings. In addition, new developments will focus on implementing advanced access control mechanisms and extending support to other data modalities, including imaging, audio, and unstructured text. These developments aim to enhance interoperability and clinical applicability while maintaining the architectural principles that ensure full traceability.

### Conclusions

This study set aimed to design and evaluate a health data platform where traceability is implemented as a central architectural feature integrated with model management, orchestration, and visualization components. DeltaTrace supports both real-time and batch processing of health-related data with built-in traceability features. In this evaluation, continuous monitoring from wearable devices has been used to assess real-time processing, while questionnaire data have been included as a representative case of batch ingestion. The evaluation demonstrated that the platform can sustain near real-time performance, processing data for approximately 1500 users with end-to-end delays below 10 minutes on a single CPU-based server. These findings confirm that the platform achieves scalable and reproducible processing under constrained computational resources.

By embedding traceability within the data lifecycle and integrating complementary components for monitoring and management, DeltaTrace addresses key requirements for auditability, reproducibility, and accountability in digital health infrastructures. The platform aligns with the objectives of the EHDS by providing a technical foundation that supports secure, version-controlled, and verifiable health data processing. The evaluation confirms that the proposed design effectively meets the defined research objectives, demonstrating that traceability, scalability, and coordinated management can be achieved within a unified and reproducible platform architecture. Future work will focus on extending DeltaTrace to strengthen privacy, interoperability, and the inclusion of additional data modalities, further aligning the platform with the long-term goals of the EHDS.

## Supplementary material

10.2196/81701 Multimedia Appendix 1 Overview of recent studies using big data capabilities in health-related applications.

10.2196/81701 Multimedia Appendix 2 Software components of the DeltaTrace platform, including Airflow, Hadoop Distributed File System, Kafka, Spark, Delta Lake, Docker, Grafana, MLflow, and PostgreSQL.

10.2196/81701 Multimedia Appendix 3 DeltaTrace platform configuration and workflow, with MLflow screenshots, Airflow directed acyclic graphs, and example configuration files for data ingestion and processing.

10.2196/81701 Multimedia Appendix 4 Results of artificial intelligence–based time-series modeling for anomaly detection in wearable data, comparing baseline, machine learning, and deep learning models with mean absolute error and root mean squared error metrics.

## Abbreviations

ACID: atomicity, consistency, isolation, and durability
AI: artificial intelligence
API: application programming interface
AttentionLSTM: attention-based long short-term memory
CPU: central processing unit
DAG: directed acyclic graph
EHDS: European Health Data Space
ELT: extract, load, transform
ETL: extract, transform, load
GPU: graphics processing unit
HDFS: Hadoop Distributed File System
ML: machine learning
PSQL: PostgreSQL
REST: Representational State Transfer
STAI: State-Trait Anxiety Inventory
