# Supplementary material for: Scalable Big Data Platform With End-to-End Traceability for Health Data Monitoring in Older Adults: Development and Performance Evaluation
Source: JMIR Med Inform. 2025 Dec 22;13:e81701. doi: 10.2196/81701 (PMC12721222; doi:10.2196/81701)
Supplement: Multimedia Appendix 1 [file medinform-v13-e81701-s001.docx]

This multimedia appendix provides a comparative overview of recent big data platforms and studies applied to healthcare domains. The purpose of this summary is to contextualize DeltaTrace within the current state of the art by highlighting the key capabilities addressed by existing works, including scalability, real-time analytics, and distributed processing. Each referenced study was analyzed in terms of data sources, computational features, and target application areas.

Table S1. Overview of recent studies utilizing big data capabilities in health-related applications. Capabilities include: Versioning (data version control), Scalability (scalability testing and handling large-scale data), Model Management (machine learning model management), Monitoring (system/resource monitoring), Scheduling (event or workflow scheduling), Storage (distributed data storage), Processing (distributed data processing), Real-time (real-time analytics support), and Visualization (built-in data visualization tools). The final columns denote the primary Task performed (e.g., classification, anomaly detection) and the Purpose or target condition addressed (e.g., diabetes, general monitoring).

|  | | | **Big Data Capabilities** | | | | | | | | | | | | | | | | **Target** | | | |  |
| --- | --- | --- | --- | --- | --- | --- | --- | --- | --- | --- | --- | --- | --- | --- | --- | --- | --- | --- | --- | --- | --- | --- | --- |
| **Reference** | **Year** | **Data** | Versioning | Scalability | | Model Management | | Monitoring | | Scheduling | | Storage | | Processing | | Real-Time | | Visualization | **Task** | | **Purpose** | |  |
| [1] | 2025 | Tweets |  | |  | |  | |  | | ✓ | | ✓ | ✓ | ✓ | | ✓ | | | Classification | | Sentiment analysis | |
| [2] | 2024 | Hospital  Demographics |  | |  | |  | |  | |  | |  | ✓ | ✓ | | ✓ | | | Classification | | Diabetes | |
| [3] | 2024 | Vital signs  Lab results |  | |  | |  | |  | |  | |  | ✓ | ✓ | |  | | | Classification | | Mortality | |
| [4] | 2023 | Tweets  UCI data |  | | ✓ | |  | |  | |  | | ✓ | ✓ | ✓ | | ✓ | | | Classification | | Heart disease | |
| [5] | 2022 | Wearables  Disease datasets |  | |  | |  | |  | |  | |  | ✓ | ✓ | | ✓ | | | Classification | | Heart disease  Diabetes | |
| [6] | 2022 | Wearables |  | |  | |  | |  | |  | |  | ✓ | ✓ | |  | | | Classification | | Breast cancer | |
| [7] | 2021 | Wearables  Questionnaires |  | | ✓ | | ✓ | | ✓ | |  | | ✓ | ✓ | ✓ | | ✓ | | | General | | General | |
| [8] | 2021 | IoT sensors |  | |  | |  | |  | |  | | ✓ | ✓ | ✓ | |  | | | Classification  Anomaly | | Diabetes | |
| [9] | 2020 | Patient data  Vital signs |  | |  | |  | |  | |  | | ✓ | ✓ | ✓ | |  | | | General | | Multi-disease | |
| [10] | 2019 | Wearables |  | |  | |  | |  | |  | | ✓ | ✓ | ✓ | |  | | | General | | General | |
| **DeltaTrace (ours)** | **2025** | **Smartwatch**  **Questionnaires** | ✓ | | ✓ | | ✓ | | ✓ | | ✓ | | ✓ | ✓ | ✓ | | ✓ | | | **General**  **Anomaly** | | **General** | |

# References

1. Ismail Azlan, Sazali Faris Haziq, Jawaddi Siti Nuraishah Agos, Mutalib Sofianita. Stream ETL framework for twitter-based sentiment analysis: Leveraging big data technologies *Expert Systems with Applications.* 2025;261:125523.
2. Saeed Mohammed A, Saeed Mogeeb A. Real-Time Diabetes Detection Using Machine Learning and Apache Spark in *2024 4th International Conference on Emerging Smart Technologies and Applications (eSmarTA)*:1–6IEEE 2024.
3. Saleh Hager, McCann Michael, El-Sappagh Shaker, Breslin John G. TransformerFusionNet: A Real-Time Multimodal Framework for ICU Heart Failure Mortality Prediction Using Big Data Streaming in *2024 International Conference on Computer and Applications (ICCA)*:1–6IEEE 2024.
4. Ed-daoudy Abderrahmane, Maalmi Khalil, El Ouaazizi Aziza. A scalable and real-time system for disease prediction using big data processing *Multimedia Tools and Applications.* 2023;82:30405–30434.
5. Yıldırım Emre, Çalhan Ali, Ciciog˘lu Murtaza. Performance analysis of disease diagnostic system using IoMT and real-time data analytics *Concurrency and Computation: Practice and Experience.* 2022;34:e6916.
6. Ebada Ahmed Ismail, Elhenawy Ibrahim, Jeong Chang-Won, Nam Yunyoung, Elbakry Hazem, Abdelrazek Samir. Applying Apache Spark on Streaming Big Data for Health Status Prediction. *Computers, Materials & Continua.* 2022;70.
7. Bahmani Amir, Alavi Arash, Buergel Thore, et al. A scalable, secure, and interoperable platform for deep data-driven health management *Nature communications.* 2021;12:5757.
8. Rashid Mamoon, Singh Harjeet, Goyal Vishal, Parah Shabir Ahmad, Wani Aabid Rashid. Big data based hybrid machine learning model for improving performance of medical Internet of Things data in healthcare systems in *Healthcare Paradigms in the Internet of Things Ecosystem*:47–62Elsevier 2021.
9. Zheng Xuan, Ding Xiaopan. Research on medical big data of health management platform based on Hadoop in *2022 21st International Symposium on Distributed Computing and Applications for Business Engineering and Science (DCABES)*:38– 41IEEE 2022.
10. Tu Yongqiu, Lu Yiqiang, Chen Guohua, Zhao Jie, Yi Faling. Architecture design of distributed medical big data platform based on spark in *2019 IEEE 8th Joint International Information Technology and Artificial Intelligence Conference (ITAIC)*:682–685IEEE 2019.
