# Supplementary material for: Scalable Big Data Platform With End-to-End Traceability for Health Data Monitoring in Older Adults: Development and Performance Evaluation
Source: JMIR Med Inform. 2025 Dec 22;13:e81701. doi: 10.2196/81701 (PMC12721222; doi:10.2196/81701)
Supplement: Multimedia Appendix 2 [file medinform-v13-e81701-s002.docx]

# Appendix 2. Open-Source Components and Versions

In this appendix, the open-source technologies used in the development of DeltaTrace are listed along with their respective versions. Providing this information ensures reproducibility, facilitates maintenance, and allows verification of the platform configuration.

- **Apache Airflow** (v2.6.3) [1] as the orchestrator for ELT pipelines, providing a robust and flexible framework for managing complex data workflows.
- **Apache Hadoop File system (HDFS)** (v3.2.1) [2] as the scalable storage backend for Delta Lake.
- **Apache Kafka** (v2.8.1) [3] for handling high-throughput data streams.
- **Apache Spark** (v3.4.0) [4] for distributed data processing across ingestion, cleaning, aggregation, and model interaction.
- **Delta Lake** (v2.4.0) by Databrick [5] for implementing the medallion architecture with built-in support for version control, ACID transactions, and schema enforcement.
- **Docker** (24.0.6) [6] to simplify platform deployment, allowing for efficient use of system resources and consistency across development and release cycles.
- **Grafana** (v9.5.3) [7] as the primary tool for generating dynamic and engaging data visualization charts.
- **MLflow** (v2.8.0) [8] for providing model traceability, experiment management, model serving and monitoring, as well as pipeline versioning.
- **PostgreSQL** (v15.4) [9]: A relational database management system used to store structured data outputs and analytics, enabling efficient querying and integration with external applications.

**References**

[1] Harenslak Bas P, De Ruiter Julian. *Data pipelines with apache airflow*. Simon and Schuster 2021.

[2] Dhulavvagol Praveen M, Totad SG. Performance enhancement of distributed system using HDFS federation and sharding *Procedia Computer Science.* 2023;218:2830–2841.

[3] Garg Nishant. *Apache kafka*. Packt Publishing 2013.

[4] Zaharia Matei, Xin Reynold S, Wendell Patrick, et al. Apache spark: a unified engine for big data processing *Communications of the ACM.* 2016;59:56–65.

[5] Haelen Bennie, Davis Dan. *Delta Lake: Up and Running: Modern Data Lakehouse Architectures with Delta Lake*. " O’Reilly Media, Inc." 2023.

[6] Miell Ian, Sayers Aidan. *Docker in practice*. Simon and Schuster 2019.

1. Chakraborty Mainak, Kundan Ajit Pratap. Grafana in *Monitoring cloud-native applications: Lead agile operations confidently using open source software*:187– 240Springer 2021.
2. Juba Salahaldin, Vannahme Achim, Volkov Andrey. *Learning PostgreSQL*. Packt Publishing Ltd 2015.
3. G Usha M, S Shreya M, S Supreeth, G Shruthi, Pruthviraja Dayananda, Chavan Pundalik. Kidney Tumor Detection Using MLflow, DVC and Deep Learning in *2024 Second International Conference on Advances in Information Technology (ICAIT)*;1:1-7 2024.
