# Supplementary material for: Scalable Big Data Platform With End-to-End Traceability for Health Data Monitoring in Older Adults: Development and Performance Evaluation
Source: JMIR Med Inform. 2025 Dec 22;13:e81701. doi: 10.2196/81701 (PMC12721222; doi:10.2196/81701)
Supplement: Multimedia Appendix 3 [file medinform-v13-e81701-s003.docx]

# Appendix 3. DeltaTrace Platform Configuration

In this appendix, the configuration of several open-source technologies included in DeltaTrace are described in greater detail. Figure S1 presents a screenshot of the MLflow web interface displaying the recorded models for the anomaly detection task applied to sleep time series collected from the wearable device. The model undergoes monthly retraining as new data are gathered, and each iteration is tagged with a version label (e.g., Version 1). If the new model achieves better performance than the latest version in terms of Mean Absolute Error (MAE), the previous version is moved to the Archived stage, and the updated model is promoted to the Production stage. MLflow also provides access to detailed information for each version, including model performance metrics, code version, and architecture.

Automatic model retraining for each data source is managed through one of four Directed Acyclic Graphs (DAGs) defined in the Apache Airflow service of DeltaTrace. These DAGs are shown in Figure S2. The Airflow user interface enables execution and monitoring of these DAGs, displaying whether a DAG has failed, is running, or when the next execution is scheduled. DAGs can also be manually triggered, and detailed execution information is available, including the status of individual tasks and resource consumption. Each DAG is created as a Python script stored in a directory used by Apache Airflow. Most tasks within the DAGs correspond to Python scripts, PySpark scripts executed via the Apache Spark Operator, or HTTP requests executed through the Simple HTTP Operator.

Several generic scripts have been developed and can be customized through straightforward configuration files. Figure S3 shows an example configuration file for reading data from Kafka topics and storing incoming data in a Delta table at the Bronze layer without applying transformations. Dictionary keys such as “wearable_steps” represent Kafka topic names being continuously read. Values define the mapping of variables received from each topic to the schema of the target Delta table in the Bronze layer.


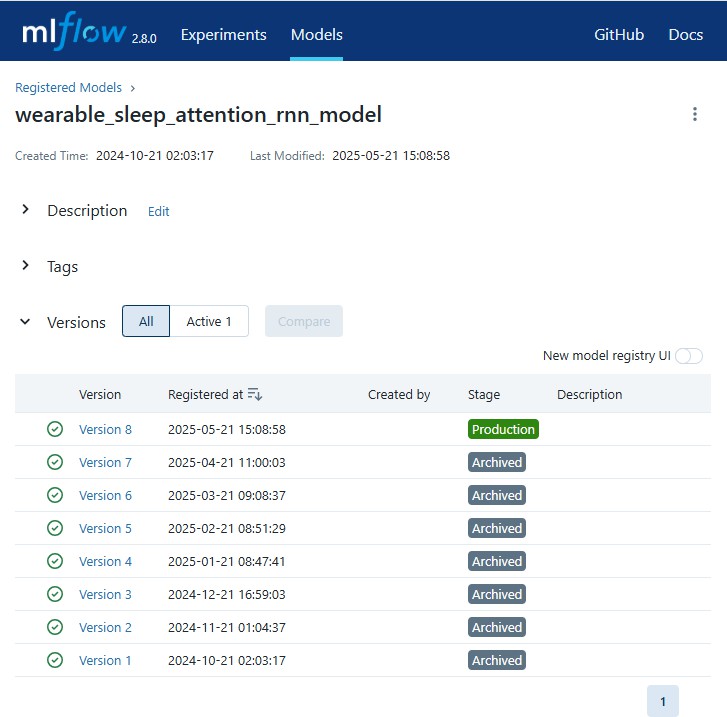


**Figure S1.** MLflow tracking interface displaying eight versions of the wearable_sleep_attention_rnn_model. The most recent version is tagged as "Production", indicating its deployment status. Each version includes metadata such as training parameters, performance metrics, and timestamps, supporting full traceability and reproducibility of the model lifecycle.


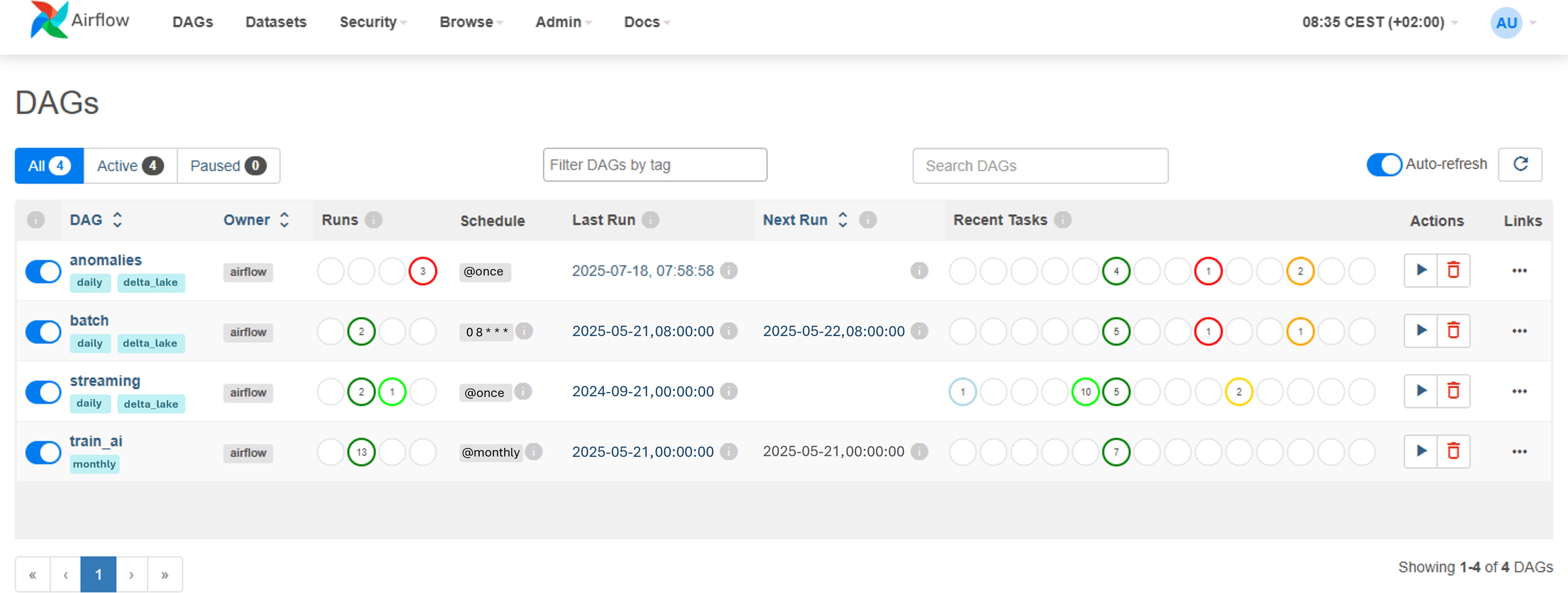


**Figure S2.** Overview of the four DAGs defined in Apache Airflow for orchestrating data and AI workflows within DeltaTrace. The anomalies and streaming DAGs operate in near real-time for continuous processing, while the batch DAG runs daily for scheduled data aggregation. The train_ai DAG executes monthly to retrain AI models using accumulated data. This modular scheduling ensures efficient resource utilization and timely execution of analytical tasks.

Similarly, Figure S4 illustrates an example configuration file defining processes that read data from a Bronze layer Delta table, apply transformations, and store the output in the Silver layer. The table name in both Bronze and Silver layers is specified under ”application_name”. The ”streaming” parameter determines whether the process runs in batch mode, while ”to_update” lists variables to be updated when duplicates are found, as specified under ”conditions”. Columns to retain are defined in ”selected_columns”. The ”group” parameter allows grouping of different processes for visualization within the corresponding Apache Airflow DAG, optimizing resource allocation.


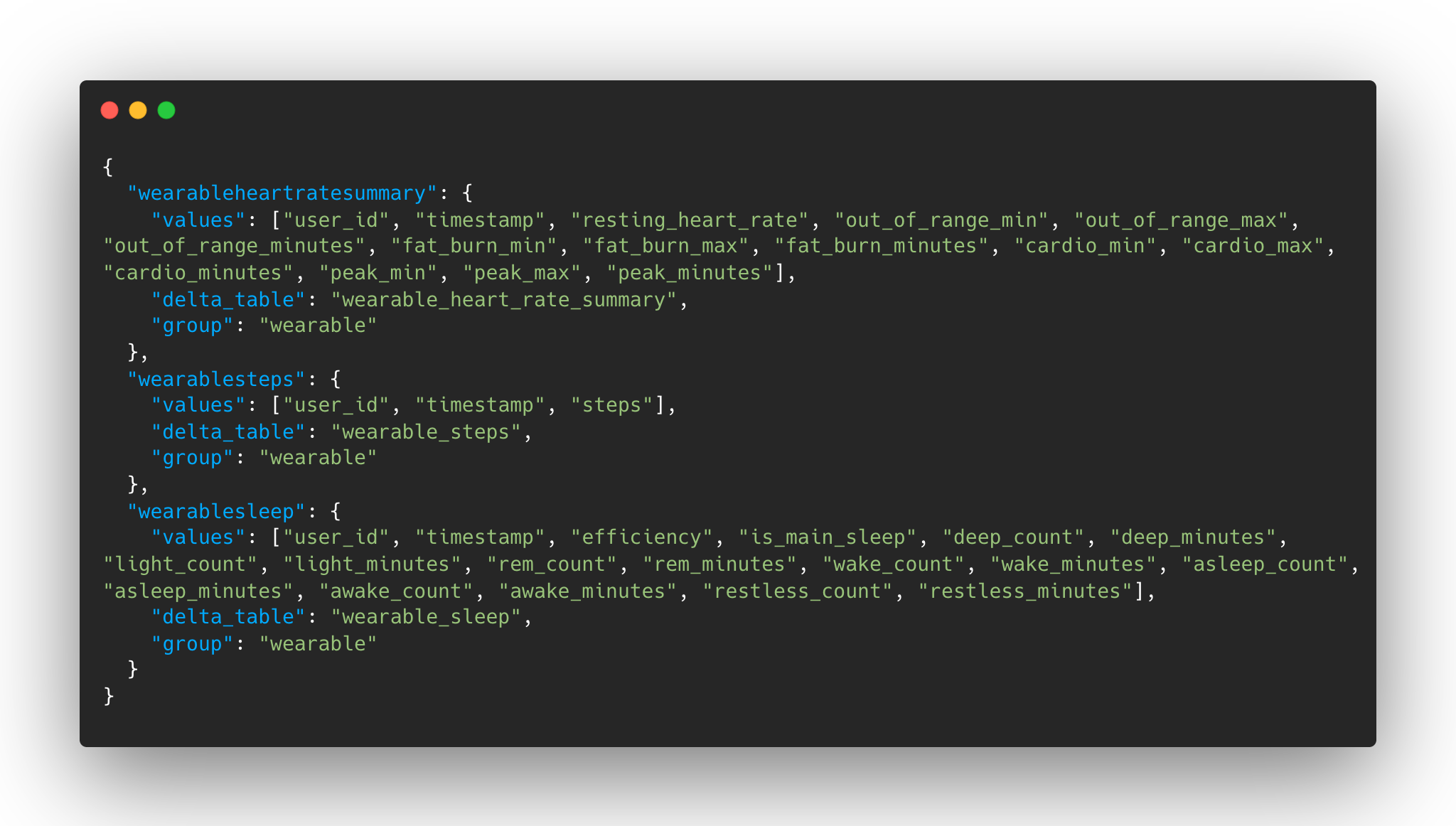


**Figure S3.** Example of a configuration file specifying Kafka topics, variable mappings, and storage in the Bronze layer.


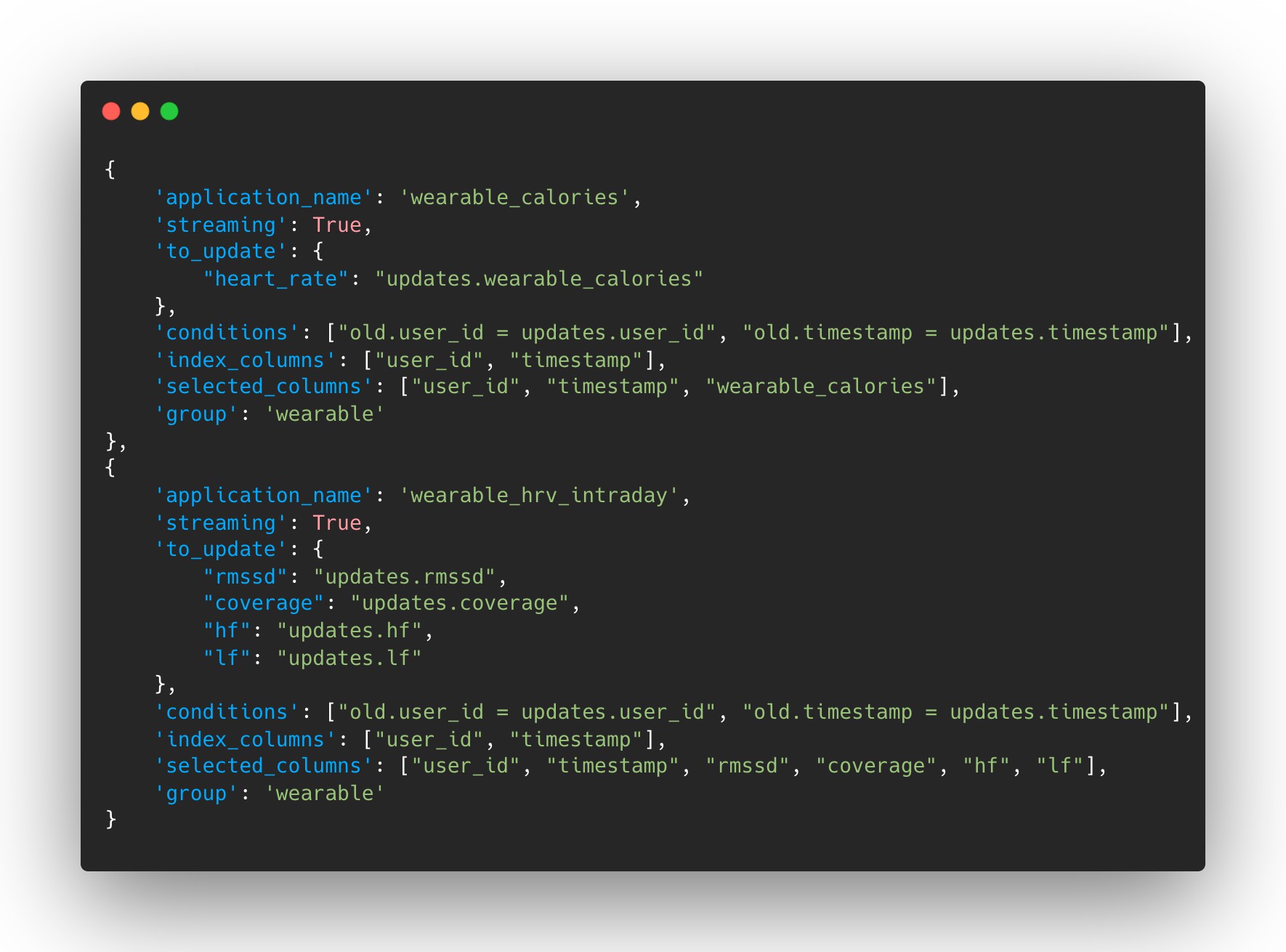


**Figure S4.** Example configuration file for loading Bronze layer data with Apache Spark Streaming, applying transformations, and storing the output in the Silver layer.
