# Supplementary material for: Scalable Big Data Platform With End-to-End Traceability for Health Data Monitoring in Older Adults: Development and Performance Evaluation
Source: JMIR Med Inform. 2025 Dec 22;13:e81701. doi: 10.2196/81701 (PMC12721222; doi:10.2196/81701)
Supplement: Multimedia Appendix 4 [file medinform-v13-e81701-s004.docx]

# Appendix 4. Results of AI-based time series modelling

The objective of this set of experiments is to evaluate the capacity of artificial intelligence models to perform next value prediction in wearable time series data from the LifeSnaps dataset, which is the basis of the anomaly detection algorithm. The detection process consists of predicting the next value in the sequence and comparing it with the actual observation. When the deviation between predicted and actual values exceeds a defined threshold, the observation is flagged as an anomaly. Consequently, the quality of the prediction model directly determines the reliability of anomaly detection.

For the benchmark, three types of approaches are included. The first is a baseline model that predicts the mean of the past values, which is the simplest way to estimate future values. The second group consists of traditional machine learning algorithms that do not explicitly account for temporal dependencies: AdaBoost, Decision Tree, K-Nearest Neighbors, and Random Forest. The third group includes deep learning models based on recurrent neural networks, which are designed to model temporal relationships in the data: LSTM, BidirectionalLSTM, ConvLSTM, and AttentionLSTM. For all deep learning models, several hyperparameters are optimized using Optuna with 30 trials. The optimized parameters include the size of the sliding window, dropout rate, batch size, the number of recurrent layers, the number of neurons in each recurrent layer, the number of convolutional layers, the size and number of convolutional kernels, and the configuration of dense layers in the output. All models are evaluated using a user-based three-fold cross validation strategy to avoid information leakage across individuals and ensure robust performance estimation.

The results in Table S1 summarize the performance of all approaches for the prediction of sleep (in hours) and steps (count). The baseline model obtains a mean absolute error (MAE) of 1.46 and a root mean squared error (RMSE) of 2.25 for sleep, and an MAE of 4208.64 and an RMSE of 5456.80 for steps. Within the machine learning group, Random Forest achieves the best performance for sleep, reducing MAE by approximately 4.15% while AdaBoost achieves the best performance for steps, reducing the MAE by 17.1% compared to the baseline. This shows that considering non-linear relationships improves predictive accuracy, although these models do not fully capture temporal structure.

Deep learning models consistently outperform the baseline and machine learning models. For sleep prediction, AttentionLSTM achieves the lowest error with an MAE of 1.33 and an RMSE of 2.12. This represents an improvement of 8.9% in MAE and 5.8% in RMSE over the baseline, and approximately 5% in MAE compared to the best machine learning model (RandomForest). For steps prediction, ConvLSTM provides the strongest results with an MAE of 3242.89 and an RMSE of 4376.29, corresponding to improvements of 22.9% and 19.8% over the baseline and 6.33% in MAE compared to AdaBoost. The superior performance of ConvLSTM for steps may be explained by its ability to integrate local spatial-temporal features through convolutional operations, which is relevant in capturing short-term variations in step count. In contrast, AttentionLSTM excels for sleep because attention mechanisms prioritize the most relevant segments of longer sequences, which is crucial for modeling the complex structure of sleep patterns.

Overall, these results confirm that deep learning models that explicitly model temporal dependencies achieve higher accuracy than machine learning approaches, which operate on aggregated features without sequential modeling. Attention mechanisms provide an additional benefit by dynamically weighting past observations, which is evident in the performance of AttentionLSTM for sleep. Based on these findings, AttentionLSTM is selected as the reference model for anomaly detection within the proposed big data platform, ensuring consistent and accurate prediction across heterogeneous data sources. This model includes two RNN layers with a built-in self-attention mechanism in the middle.

**Table S1.** Performance comparison of baseline, machine learning, and deep learning models for next value prediction in wearable time series using user-based three-fold cross-validation. Metrics include Mean Absolute Error (MAE) and Root Mean Squared Error (RMSE) for both sleep and step prediction tasks. Bold values indicate the best-performing model for each metric and task.

**Sleep Steps**

| **Model** |  | |  | |
| --- | --- | --- | --- | --- |
|  | **MAE** | **RMSE** | **MAE** | **RMSE** |
| *Baseline*  Mean | 1.46 (±0.06) | 2.25 (±0.04) | 4208.64 (±217.63) | 5456.80 (±437.97) |
| *Machine learning* AdaBoost DecisionTree KNN  RandomForest | 1.46 (±0.06)  2.03 (±0.14)  1.52 (±0.03)  1.40 (±0.04) | 2.24 (±0.09)  3.08 (±0.19)  2.30 (±0.08)  2.20 (±0.05) | 3489.72 (±167.39)  4642.07 (±370.20)  3613.84 (±252.58)  3582.14 (±143.42) | 4717.12 (±209.93)  6309.17 (±441.37)  4781.65 (±289.34)  4764.56 (±276.04) |
| *Deep learning* AttentionLSTM BidirectionalLSTM ConvLSTM  LSTM | **1.33 (±0.06)**  1.35 (±0.03)  1.35 (±0.04)  1.36 (±0.02) | **2.12 (±0.18)**  2.15 (±0.06)  2.14 (±0.08)  2.17 (±0.06) | 3646.77 (±332.19)  3649.85 (±592.64)  **3242.89 (±147.52)**  3568.52 (±614.78) | 4844.78 (±353.62)  4896.81 (±685.02)  **4376.29 (±214.20)**  4740.96 (±730.48) |
